# Head to toe ultrasound: a narrative review of experts’ recommendations of methodological approaches

**DOI:** 10.1186/s44158-022-00072-5

**Published:** 2022-10-21

**Authors:** Antonio Messina, Chiara Robba, Rita Bertuetti, Daniele Biasucci, Francesco Corradi, Francesco Mojoli, Silvia Mongodi, Eduardo Rocca, Stefano Romagnoli, Filippo Sanfilippo, Luigi Vetrugno, Gianmaria Cammarota

**Affiliations:** 1 Humanitas Clinical and Research Center – IRCCS, Rozzano (Milano), Italy; 2 Department of Biomedical Sciences, Humanitas University, Pieve Emanuele (Milan), Italy; 3 Anesthesia and Intensive Care, Ospedale Policlinico San Martino, IRCCS Per L’Oncologia E Le Neuroscienze, Genoa, Italy; 4 Dipartimento Di Scienze Chirurgiche E Diagnostiche Integrate, Università Di Genova, Genoa, Italy; 5 Department of Anesthesiology, Intensive Care and Emergency, Spedali Civili University Hospital, Brescia, Italy; 6 Department of Clinical Science and Translational Medicine, Tor Vergata’ University of Rome, Rome, Italy; 7 Emergency Department, Tor Vergata’ University Hospital, Rome, Italy; 8 Department of Surgical, Medical and Molecular Pathology and Critical Care Medicine, University of Pisa, Pisa, Italy; 9 Department of Clinical-Surgical, Diagnostic, and Pediatric Sciences, Unit of Anesthesia and Intensive Care, University of Pavia, Pavia, Italy; 10 Anestesia E Rianimazione I, Fondazione IRCCS Policlinico San Matteo, Pavia, Italy; 11 Dipartimento Di Medicina Traslazionale, Università del Piemonte Orientale, Novara, Italy; 12 Department of Health Science, University of Florence, Florence, Italy; 13 Department of Anesthesia and Intensive Care, A.O.U. “Policlinico-San Marco”, Catania, Italy; 14 Department of Medical, Oral and Biotechnological Sciences, University of Chieti-Pescara, Chieti, Italy; 15 Dipartimento Di Medicina E Chirurgia, Università Degli Studi Di Perugia, Perugia, Italy

**Keywords:** Critical Care, Point-of-care Ultrasound, Methodology

## Abstract

Critical care ultrasonography (US) is widely used by intensivists managing critically ill patients to accurately and rapidly assess different clinical scenarios, which include pneumothorax, pleural effusion, pulmonary edema, hydronephrosis, hemoperitoneum, and deep vein thrombosis. Basic and advanced critical care ultrasonographic skills are routinely used to supplement physical examination of critically ill patients, to determine the etiology of critical illness and to guide subsequent therapy. European guidelines now recommend the use of US for a number of practical procedures commonly performed in critical care. Full training and competence acquisition are essential before significant therapeutic decisions are made based on the US assessment. However, there are no universally accepted learning pathways and methodological standards for the acquisition of these skills.

Therefore, in this review, we aim to provide a methodological approach of the head to toe ultrasonographic evaluation of critically ill patients considering different districts and clinical applications.

## Introduction

Critical care ultrasonography (US) is considered an essential tool for the initial evaluation and clinical management of critically ill patients admitted to the intensive care unit (ICU), combining simple protocols of US application, a prompt assessment of critical conditions and therapeutic decisions. As a matter of fact, critical care US is nowadays considered not only a diagnostic tool providing information about the source of critical illness but can also be a component of the patients’ physical exam. This approach expands the sensitivity and specificity of clinical examination and basic measurements and is oriented to the peculiarity of the single patient.

The diffusion of critical care US has been slowed down over the last years because of technical issues (portability and availability of the machines), and lack of formal and standardized training programs. These gaps have been recently overcome by technical advances providing high-quality images at the bedside, and by the development of new guidelines for skills certification. Recently, a consensus of the European Society of Intensive Care (ESICM) provided a number or recommendations for the head to toe basic skills to be obtained by intensivists managing critically ill patients [1], and focusing on five districts (brain, lung, heart, abdomen, and vascular ultrasound).

According to that paper, the aim of this review is to provide a methodological approach of the US evaluation of different body districts and a critical reappraise of available training programs in the literature for the implementation of head to toe US in ICU.

## Methods

For the purpose of this review, we asked the contribution of ICU Italian physicians with recognized expertise in US use in the critical care setting. Criteria for panel selection included high scientific knowledge and production in the field of ultrasonography and recognized clinical expertise.

We finally involved 12 experts, who were further divided in 5 subgroups, each one addressing different districts: brain, heart, thorax, abdomen, and vessels US. The experts were asked to summarize the methodological issues related to the application of US in the ICU, and to provide a review of the available training programs and studies aimed at providing competences for the application of US in critically ill patients.

### The brain

**Fig. 1:**
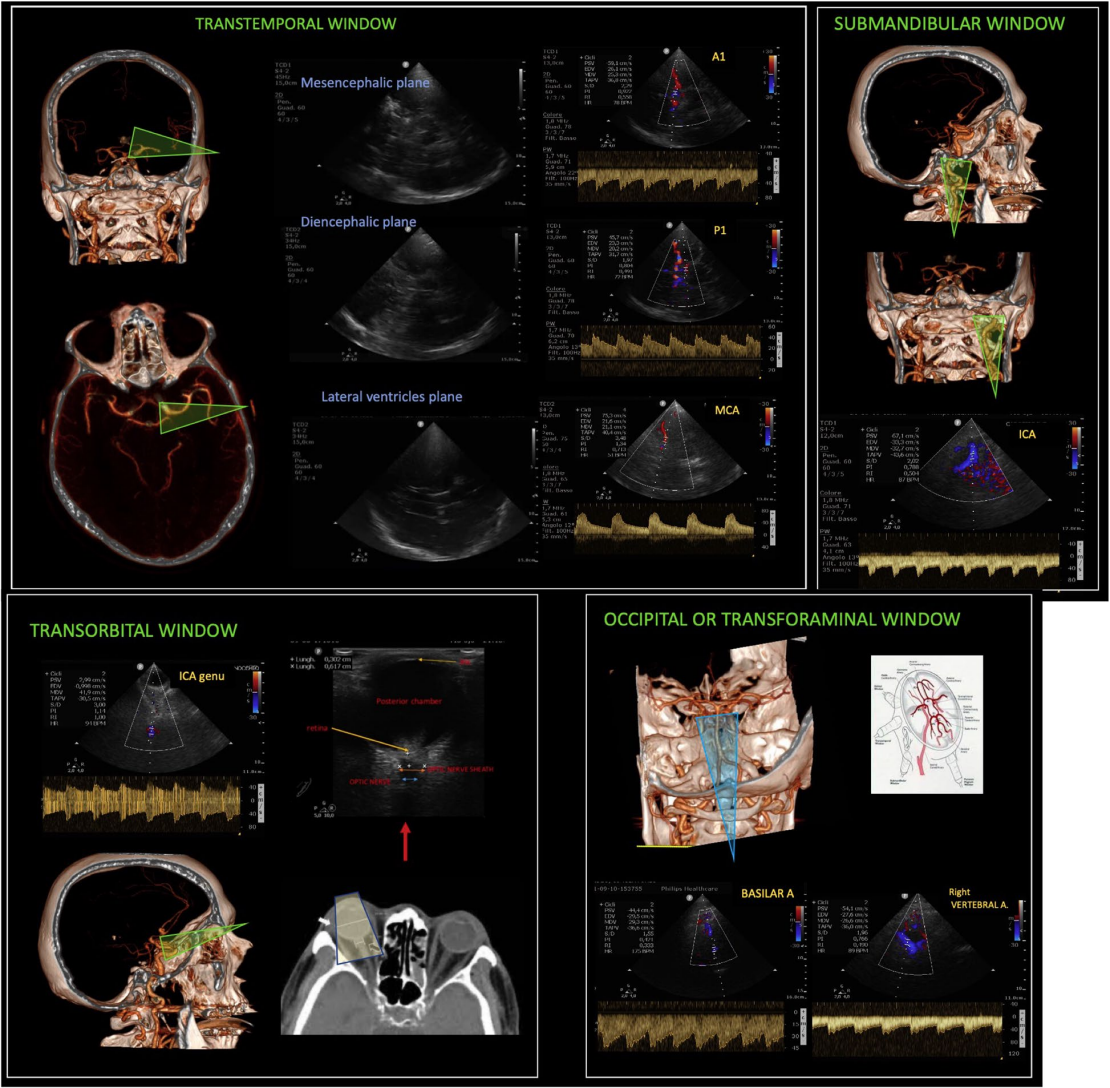
Transcranial color-coded duplex ultrasonography. The insonation windows for cerebral blood flow velocities assessment are shown. For the insonation of the anterior cerebral artery, middle cerebral artery, and posterior cerebral artery, the transtemporal window is used. For internal carotid artery, submandibular window; for basilar artery and vertebral artery, the occipital window is used. A1, anterior cerebral artery A1 tract; MCA, middle cerebral artery; P1, posterior cerebral artery P1 tract; ICA, internal carotid artery

Brain ultrasound (BUS) is getting interest as bedside tool for intensivists and emergency physicians, with an important role in the early diagnosis and management of acute intracranial pathology [2]. The use of traditional transcranial Doppler, generally limited to the neurosonology laboratories settings, has expanded over the last years with the introduction of B-mode ultrasound and color Doppler, the transcranial color-coded duplex ultrasonography (TCCD) (Table 1, Fig. 1).

**Table 1:**
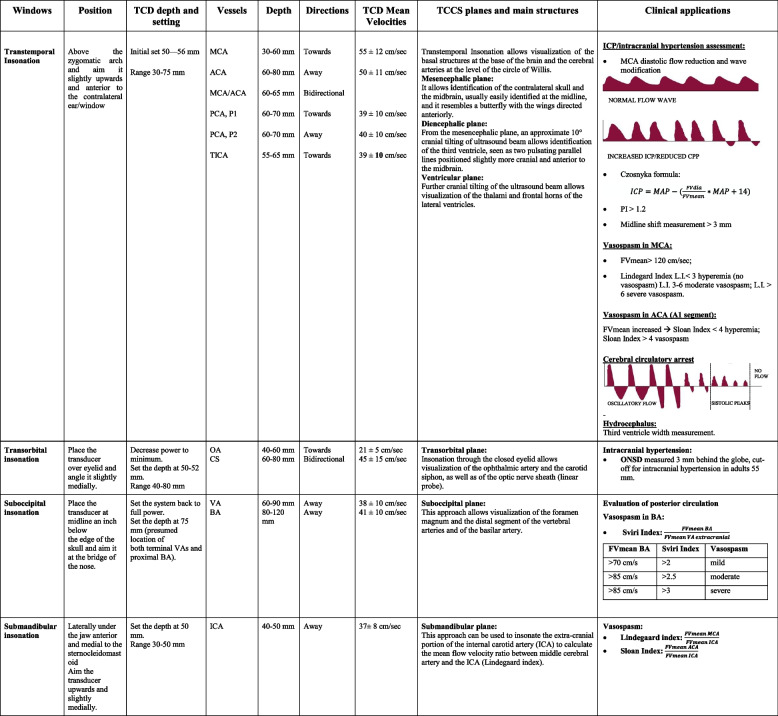
Anatomical landmarks and normal velocity values of TCD and TCCS, main clinical application

*MCA* Middle cerebral artery, *ACA* Anterior cerebral artery, *PCA* Posterior cerebral artery, *P1* first segment of the posterior cerebral artery, *P2* second segment of the posterior cerebral artery, *TICA* Terminal internal carotid artery, *CS* Carotid siphons, *VA* Vertebral artery, *BA* Basilar artery, *ICA* Internal carotid artery, *FVmean* mean flow velocity, *FVdia* diastolic flow velocity, *PI* pulsatility index, *L.I.* Lindegaard Index, *ONSD* Optic nerve sheath diameter, *ICP* Intracranial pressure, *CPP* cerebral perfusion pressure

The use of this technique has opened new possibility for the assessment of intracerebral pathophysiology, not only for neurocritical patients but also in general intensive care unit and emergency room settings [3].

Clinical indications of TCCD currently include the evaluation of cerebral anatomy (midline shift, intracerebral hemorrhage, hydrocephalus), as well as the assessment of cerebrovascular diseases. The analysis of the waveform obtained from the major intracranial vessels allows in fact the management of patients in different scenarios such as traumatic brain injury, subarachnoid hemorrhage, and neurological complications of general ICU patients such as cardiac arrest, sepsis, and respiratory failure [4].

Clinical applications of BUS include:

- The evaluation of normal or altered cerebral anatomy. In particular, TCCD is able to assess the presence of intracranial hemorrhage, midline shift, hydrocephalus, and the need for external ventricular drain [5–10].
- The use as a confirmatory test in the assessment of brain death, which is determined as oscillatory reverse flow, systolic spikes, and absence of flow [11].
- Noninvasive and indirect evaluation of intracranial pressure (ICP) and cerebral perfusion pressure, based on different techniques obtained from the flow velocity waveform analysis [12], optic nerve sheath diameter [13], and midline shift assessment. Recently, a large multicenter study [14] suggested an excellent negative predictive value for TCCD for the evaluation of ICP, thus suggesting that this tool can be used to exclude the presence of increased ICP in different clinical contexts, including the focused assessment sonography for trauma.
- Angiographic vasospasm and delayed cerebral ischemia are the most severe consequences in aneurysmal subarachnoid hemorrhage (aSAH) patients: in this context, TCCD ultrasonography has shown to be reliable even in comparison with digital subtraction angiography (DSA) in the detection of increased flow velocity in the middle cerebral artery [15].
- Cerebral autoregulation (CA) is the ability of the brain to maintain cerebral blood flow (CBF) constant despite changes in cerebral perfusion pressure: the flow velocity is directly correlated with CBF; therefore, TCD allows evaluation of both static and dynamic components of CA, including transient hyperemic response test, cuff test, and the Mx index, calculated as the correlation coefficient between flow velocity and arterial blood pressure [16, 17].

BUS is generally performed using four specific insonation windows: transtemporal, transforaminal (or suboccipital), submandibular and transorbital. A phased array 1.5–4 MHz low-frequency probe is used for the insonation of intracranial structures, while linear 7–15 MHz high frequency is used for ultrasound of optic nerve sheath.

Training paths and skills certification for the use of BUS by intensivists are still matter of debate among Scientific Societies and experts. Currently, only the American Academy of Neurology advises for the routine use of TCD in the neurocritical care setting [1]

### The lung

**Fig. 2:**
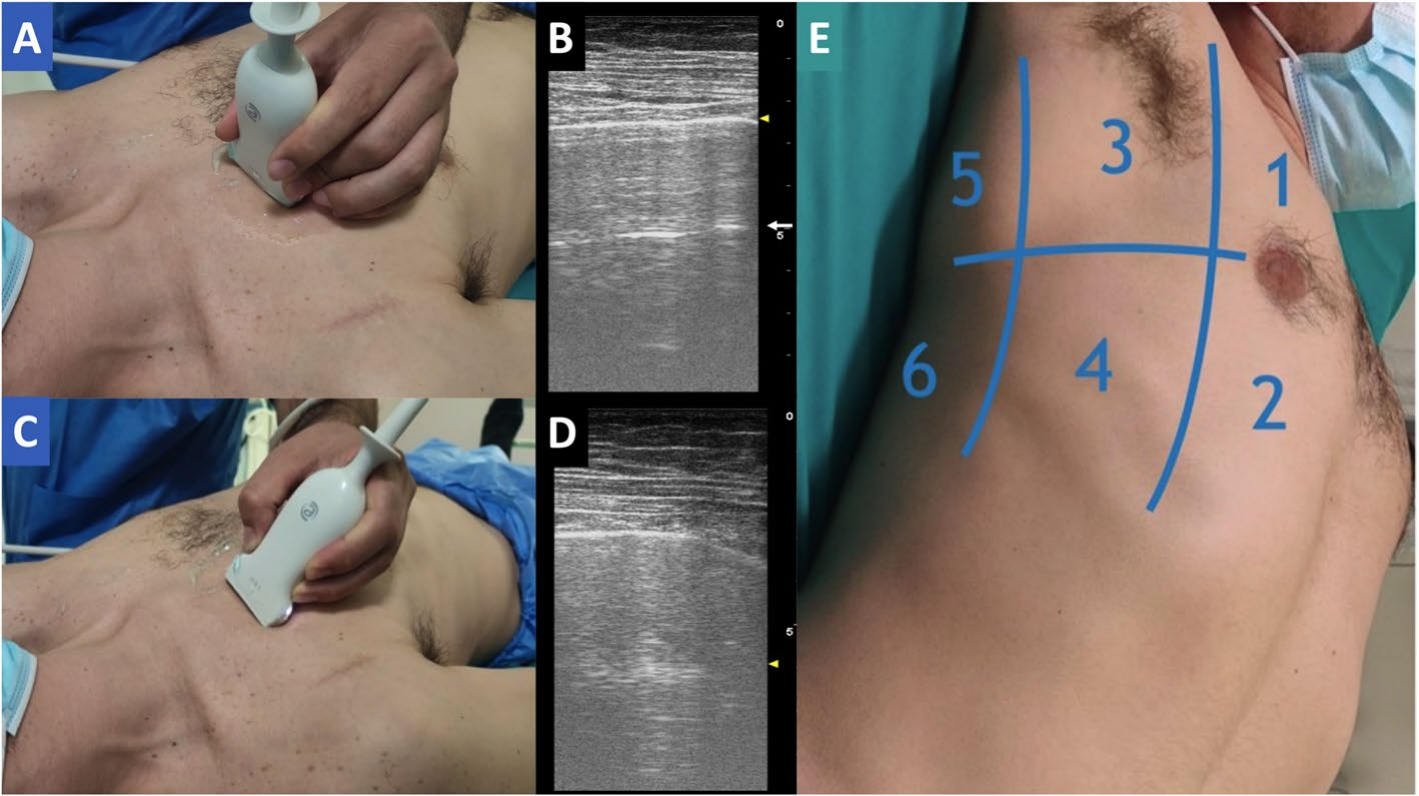
Lung ultrasound. **A** Transversal scan of an anterior intercostal space: the probe is tilted to be perpendicular to the pleura. In the corresponding ultrasound image (**B**), the pleural line and one A-line (white arrow) are well visible. One focus is correctly set on the pleura (yellow triangle). **C** In the same intercostal space, the probe is not correctly tilted and is not perpendicular to the probe. In fact, in the corresponding scan, the A-line is not visible anymore (**D**); moreover, the focus is set too deep, leading to a less distinct visualization of the pleural line. **E** The 6 regions per hemithorax of a standard lung ultrasound examination. Anterior fields (1 and 2) are identified by sternum and anterior axillary line, lateral fields (3 and 4) by anterior and posterior axillary lines, posterior fields are accessible below the posterior axillary lines (5 and 6). The patient can lie supine with no need to turn him/her to visualize posterior fields

Lung ultrasound gained a leading position in critical care both as a diagnostic and a monitoring tool [18–20], facing a large spread during coronavirus 2019 disease (COVID-19) pandemic [21, 22]. It is now considered part of the basic core competences for all intensivists [23]. Different approaches in terms of probe, scan, and number of examined regions are found in literature [24–27]. Different types of examinations in the critically ill have been proposed. A complete and systematic one includes the evaluation of 6 regions per hemithorax [24–27]: anterior, lateral, and posterior fields which are identified by sternum, anterior and posterior axillary lines, respectively, and each field is divided into superior and inferior regions (Fig. 2E). In case lung ultrasound is performed to rule in/out a specific diagnosis, especially in emergency, we suggest performing a focused approach to answer the specific question. For example, to rule out pneumothorax in supine thoracic trauma, one scan per side in anterior fields will be enough [28].

Although a significant difference in probes performance could not be found in children [29], we suggest starting in anterior fields with a linear probe, as this is the one validated with quantitative computed tomography (CT) scan [30]. In modern machines, it is important to abolish artifact-erasing software and harmonics to optimize the artifacts’ visualization [31]. The switch to a low-frequency probe is useful in case consolidations/pleural effusions are visualized or if the chest wall is particularly thick. For lung US examination, the probe can be oriented longitudinally (i.e., aligned with craniocaudal axis) or transversally (i.e., aligned with intercostal space). A longitudinal approach allows the identification of the bat sign and therefore of the intercostal space; it is recommended for beginners or in any case of difficult examination (for example: subcutaneous emphysema making unclear the pleural line visualization). A transversal approach allows the visualization of wider pleura [32] but requires higher expertise; it is recommended for the measure of interpleural distance and the quantification of pleural effusions [28]. With any approach, once the pleural line identified, the probe has to be tilted searching for the A-lines, reverberation artifacts visible when the probe is well-oriented perpendicular to the pleura: they can be considered a marker of good-quality images (Fig. 2A–D).

Bi-dimensional images can be associated to M-mode, particularly useful to confirm the presence/absence of lung sliding (respectively visualized with seashore and stratosphere signs), thanks to its higher frame rate.

Color Doppler is rarely used in lung ultrasound; it can help identifying shunt in consolidated lobes [33], although this assessment is purely qualitative so far; it has recently been proposed also for the diagnosis of ventilator-associated pneumonia [34].

Automation has been proposed to facilitate the interpretation of lung ultrasound in the critically ill [35–37]: it showed good correlation with extra-vascular lung water and good diagnostic accuracy for cardiogenic/non-cardiogenic lung edema and COVID-19 pneumonia; however, so far visual analysis of the expert remains the most reliable tool.

A training curriculum of 25 supervised examinations resulted adequate to acquire basic skills in lung ultrasound [38]; however, the exact definition of training for specific signs as air-bronchogram is still matter for research [39]. Finally, quantitative approach for the assessment of lung aeration should not be considered a basic skill [1] and requires a dedicated training [40].

### The heart

The advent of portable ultrasound machines has revolutionized the clinical application of cardiac US, allowing physicians to assess cardiovascular complications at the patient’s bed. In particular, cardiac ultrasound with transesophageal echocardiography (TEE), first used in 1981, was moved from the necessity to evaluate the surgical result immediately and monitor the hemodynamic state of the patients with post-cardiotomy shock in terms of fluid and inotrope in the operating room [41]. That led to the publication of TEE practice perioperative guidelines in 1996 and the development of cardiovascular societies worldwide of many courses for training and practice in cardiac anesthesia (the first TEE examination took place in 1998 and rapidly became the international standard) [42, 43]. That has initiated the era of point-of-care ultrasound (POCUS). However, POCUS did not begin until 1990, when more compact machines were available.

Developing and applying echocardiography within the critical care setting required another 10 years since the American College of Chest Physicians and La Société de Reanimation de Langue Française in 2009 published a consensus statement to describe how the use of ultrasound should be considered and described what type of skills should be mastered to reach competence[44]. This panel divided the use of ultrasound in two main branches: the general critical care ultrasound with focus on the assessment at thorax, abdominal, and vascular level, and the critical care echocardiography (CCE) with two levels of skill, basic and advanced. In particular, basic CCE was intended as the use of ultrasound to perform a “goal-directed examination” with a simple question in the context of clinical syndromes. For instance, questions are “is my shocked patient having cardiac tamponade?” or “have my shocked patients a severely reduced systolic function?”, with a qualitative rather than quantitative assessment. Therefore, basic CCE essentially includes 2D and some M-mode imaging, with limited anatomical or functional quantification parameters.

In contrast, advanced CCE refers to a more comprehensive hemodynamic assessment that requires quantitative evaluation of cardiovascular function (without or with TEE use). For example, in patients with shock, advanced assessment would refer to the quantification of left ventricular ejection fraction, evaluation of diastolic dysfunction and valvular diseases. In this context, color and spectral Doppler modes are applied with the aim to provide an appreciation of patho-physiological conditions.

Considering that the transthoracic echocardiography (TTE) percentage of successful diagnostic exams in the ICU is approximately 50% vs over 90% in the elective cardiac lab, and also that an adequate TTE window is obtained in 36% as compared to 97% with TEE, even if the main focus for echocardiography in ICU remain training in TTE, it must be considered also the importance of achieving education in TEE as well [45].

**Fig. 3:**
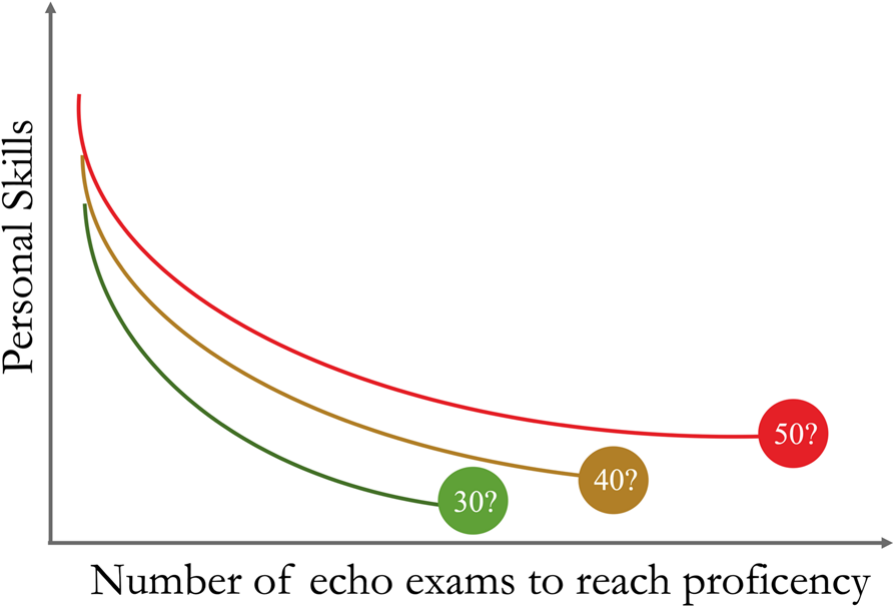
Learning curves required for the achievement of critical care echocardiography skills. Thresholds of 30, 40, and 50 examinations have been proposed

For training in CCE, an experts panel representing 13 Scientific Societies of Critical Care has recently determined the basic and advanced skills (Fig. 3). After that, many protocols have been developed worldwide to solve a specific question of monitoring fluid status or cardiac function or a specific situation like cardiac arrest, as the case of the RUSH protocol, also included in the ACLS [46, 47]. Although the step size from basic and advanced cardiac ultrasound is significant, there is a continuum in between. The American College of Cardiology and the American Heart Association documents have provided important knowledge on the cognitive skills required for basic echocardiography [48]. In daily practice, basic CCE skills can have a potentially significant clinical impact on traumatized patients and in case of emergencies requiring a rapid response and differential diagnosis. On the other hand, advanced CCE plays a growing role in cardiac anesthesia and ICU. Of note, availability of online educational material has grown exponentially with smartphones and social media such as Twitter. Also, echocardiography simulators have contributed in expanding training opportunities (especially for TEE use), accelerating at least the image acquisition process. As a result, basic, intermediate, and advanced certifications in TEE for physicians routinely or not practicing cardiac anesthesia are now available (Table 2).

**Table 2:** Cardiac ultrasound training pathways

Accreditation	*N* of scans	Written and practical exams; course attendance	Website info
**BASIC LEVEL**			
**FEEL**	50	No exams, 1-day course attendance	https://www.resus.org.uk/
**FUSIC – Heart**	50	No exams, 1-day course attendance	https://www.ics.ac.uk/
**CICM**	30	Written exam only, CICM course attendance	https://www.cicm.org.au/Trainees/Training-Courses/Focused-Cardiac-Ultrasound
**ANZCA**	40	Written exam only^A^	https://www.anzca.edu.au/education-training
**NBE Basic Perioperative TOE**	50	Written exam and 150 scans interpreted	https://www.echoboards.org/
**INTERMEDIATE LEVEL**			
**BSE Level 1**	75	Practical exam only	https://www.bsecho.org/
**ADVANCED / EXPERT LEVEL**			
**BSE ACCE**	250^B^	Written and practical exam	https://www.bsecho.org/
**BSE TOE**	125^C^	Written and practical exam	https://www.bsecho.org/
**EACVI TOE**	125^D^	Written and practical exam	https://www.escardio.org/Sub-specialty-communities/European-Association-of-Cardiovascular-Imaging-(EACVI)
**EDEC (TOE and TTE)**	100 TTE 35 TOE	Written and practical exam, course attendance and intensivist diploma	https://www.esicm.org/education/edec-2/
**NBE Advanced Perioperative TOE**	150	Written exam, cardiothoracic anesthesia fellowship (1-year and 300 scans interpreted)	https://www.echoboards.org/
**NBE CCE**	150	Written exam, 1-year ICU training dedicated to CCE (or 750 h ICU experience)	https://www.echoboards.org/
**ANZCA**	200	Written exam^E^	https://www.anzca.edu.au/education-training
**CICM Advanced**	450	Written exam^F^ (and additional 50 TOE for combined accreditation)	https://www.cicm.org.au/Trainees/Training-Courses/Focused-Cardiac-Ultrasound
**CICM Expert**	-	7 years advanced CCE practice, education, training, or research experience	https://www.cicm.org.au/Trainees/Training-Courses/Focused-Cardiac-Ultrasound

List of several critical care and/or transoesophageal echocardiography training pathways

*ICU* intensive care Unit, *CCE* critical care echocardiography, *TTE* transthoracic echocardiography, *TOE* transoesophageal echocardiography

^A^demonstrated by university post-graduate certificate; ^B^125 scans if BSE or EACVI TTE/TOE accredited; ^C^75 scans if BSE or EACVI TTE/TOE accredited; ^D^75 scans if EACVI TTE certified; ^E^demonstrated by university post-graduate diploma, NS NBE, BSE, or EACVI; ^F^demonstrated by completion of nationally or internationally recognized exit examination

Similar echocardiography disciplines prove that the learning curve for basic CCE may flatten out after 30 supervised studies [49]. The American College of Cardiology and the American Heart Association recommended that anesthesiologists perform at least 50 supervised examinations to reach competence in perioperative TEE, in conjunction with mastering mandatory cognitive skills [48]. Others found that the number of minimum supervised TEE examinations predicting 100% competence is still around 50 scans [50]. However, as “a number” itself derived from a monitoring tool cannot guarantee competence and clinical management, this should be integrated with other physiological and clinical factors.

### The diaphragm

Diaphragm ultrasound has been increasingly employed in daily clinical practice and research to assess diaphragmatic function both in and outside ICU [1, 51–57]. Recently, the evaluation of diaphragmatic excursion through ultrasonography during weaning from invasive mechanical ventilation (IMV) has been recommended as a basic skill for ICU physicians [1]. Despite this increasing interest, there is a wide variability in diaphragmatic ultrasound methodology that makes difficult to draw definite conclusion on the clinical and research impact of this tool at bedside. To enhance the standardization of diaphragmatic ultrasound assessment in the critical care setting, an international experts’ consensus statement has been just published [51]. The sonographic evaluation of the diaphragm involves the assessment of diaphragmatic muscle mass inferred by the measurement of muscular thickness as well as its changes over time, i.e., the diaphragmatic contractile activity as described by thickening fraction, and excursion during active breath [58–60]. Diaphragmatic excursion has been employed to predict noninvasive ventilation response in patients admitted to emergency department for hypoxic-hypercapnic respiratory failure [56]. Advanced respiratory monitoring of diaphragmatic thickness and thickening fraction have been used in patients requiring noninvasive respiratory support for acute respiratory failure related or not to coronavirus 2019 disease [61], in- and outside the ICU [52, 62, 63]. More recently, the assessment of diaphragmatic thickness and thickening fractions have shown to be helpful to ascertain the development of IMV-induced diaphragmatic atrophy as well as to predict IMV liberation [64] (Table 3).

**Table 3:** Diaphragmatic ultrasound

Parameter	Setting	Target	Cut-off values	Number of patients (*n*)
Excursion	Healthy subjects	Normal values	Quiet breathing: 0.9–1 cm Voluntary sniffing: 1.6–1.8 cm Deep breathing: 3.7–4.7 cm	(210)
		Dysfunction	< 2 cm	
	ICU-IMV	Successful extubation	> 1.1 cm	(52)
	ICU-IMV	Dysfunction	< 11 mm	(34)
Thickness	Healthy subjects	Normal values	1.8–3.0 mm A side-to-side difference in thickness at end expiration of > 0.33 cm is abnormal Minimally affected by age, gender, body habitus, or smoking history	(150) (109)
		Successful extubation	> 1.7	(63)
	Covid-19 spontaneously breathing	Need of IMV	2.2 mm	(77)
	ICU-IMV	Diaphragmatic weakness	2.4 ± 0.8 mm	(107)
	ICU-IMV	Atrophy	1.9 ± 0.4 mm	(54)
	ICU	Atrophy	Reduction > 10% within the first 5 days	(97)
Thickening fraction	Healthy subjects	Normal values with tidal breathing	20% ± 16% right side 23.5% ± 24.4 left side	(150)
	ICU COVID-19 CPAP	CPAP failure	21%	(27)
	ICU-IMV	Successful SBT	36%	(46)
	ICU-IMV	Successful weaning	26%	(34)
	ICU-IMV	Diaphragm dysfunction; Length of IMV	29%	(112)
	ICU-IMV	Successful extubation	34%	(52)
	ICU-IMV	Successful extubation	20%	(56)
	ICU-IMV	Successful extubation	30%	(63)
Diaphragmatic Kinetics by TDI	ICU-IMV	Extubation failure	Inspiratory peak velocity: ≥ 3.1 cm/s Inspiratory mean velocity: ≥ 1.6 cm/s Inspiratory acceleration time: ≥ 8.8 cm/s^2^ Peak relaxation velocity: > 2.6 cm/s Expiratory mean velocity: ≥ 1.1 cm/s Expiratory acceleration time: ≥ 11.2 cm/s^2^	(100)

*COVID-19* Coronavirus disease 2019, *CPAP* continuous positive airway pressure, *ICU* intensive care unit, *IMV* invasive mechanical ventilation, *SBT* spontaneous breathing trial, *TDI* tissue Doppler imaging

**Fig. 4:**
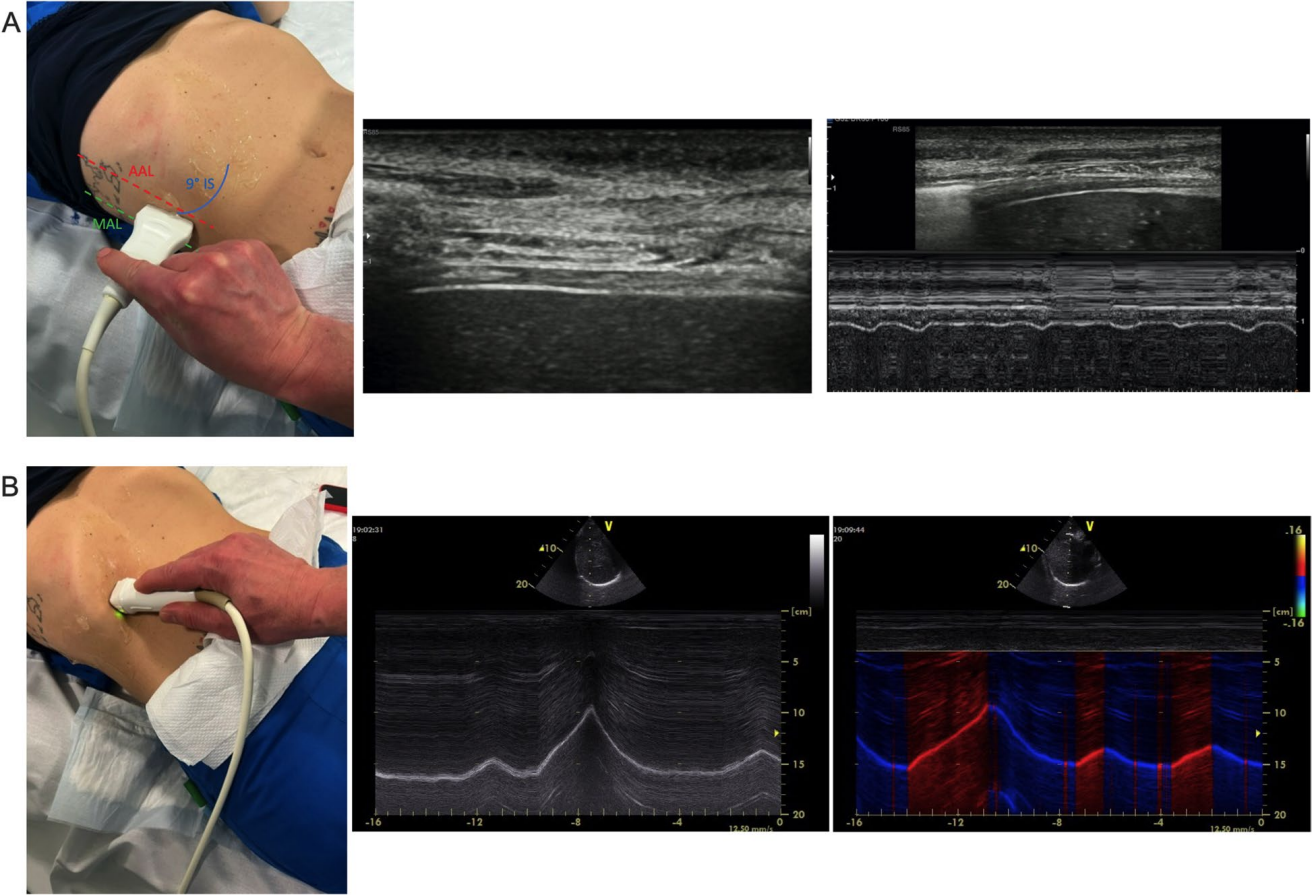
Diaphragmatic ultrasound. Diaphragmatic ultrasound for thickness (**A**) and excursion (**B**) are depicted. **A** Diaphragmatic is described as a three-layers structure involving a hypoechoic stratum between two hyperechoic edges, i.e., external pleural line and internal peritoneal lines; diaphragmatic thickness changes at varying phases of respiratory cycle. **B** Diaphragmatic displacement variations in response to respiratory cycle. Red, inspiratory excursion; Blue, expiratory excursion

As depicted in Fig. 4A, the ultrasound evaluation of diaphragmatic excursion is conducted in M-mode by directing a 2–5 MHz sectorial/convex probe towards the dome of the diaphragm [51]. The depth is adjusted to catch the maximum excursion of the diaphragm paying attention to set the gain in order to obtain the right contrast with surrounding structures. The sonographic evaluation of diaphragmatic thickness is obtained using a 7–12-MHz linear transducer, perpendicularly placed on the chest area delimited by the middle and anterior axillary lines and by the 8th and 11th ribs (Fig. 4B). The probe is indiscriminately positioned in line with or perpendicular to the intercostal space. The diaphragmatic thickness evaluation is conducted in the zone of apposition of the diaphragm to the chest wall, with the lung slightly appearing into the image. The diaphragm is described as a three-layer structure, i.e., peritoneal, fibrous, and pleural line from the liver surface to the chest wall. The gain is optimized to obtain the sufficient contrast with surrounding structures in B- or M-mode. Diaphragmatic thickness is measured positioning the caliper as close as possible to the pleural and peritoneal edge, excluding these lines by measurements. Diaphragmatic displacement and thickness acquired on the right side reflect the activity of the whole diaphragm in absence of unilateral disease. The placement of a cutaneous marker on the area selected for diaphragmatic ultrasound has demonstrated to enhance reliability as well as the intra- and inter-rater agreement of diaphragmatic sonographic assessment [51].

Normal values of diaphragmatic displacement, thickness, and thickening fraction along with the measurements indicative for diaphragmatic atrophy and dysfunction are reported in Table 3 [51, 65].

The ultrasound report should always include mechanical ventilator settings (including the applied pressure support and positive end expiratory pressure), patient positions (recumbent, semi-recumbent, sitting or standing), and previous comorbidities (e.g., chronic obstructive pulmonary diseases or myopathies) because all these factors possibly affect the measurements of diaphragmatic muscle mass and function.

In the absence of ultrasound experience, the learning curve for gaining sufficient skills to conduct a diaphragmatic ultrasound assessment for thickness and displacement in clinical practice requires at least 40 bilateral examinations, of which 20 under the expert supervision [57], even if this threshold does not consider the previous ultrasound experience of the practitioner. However, its authors’ opinion that diaphragmatic ultrasonography should always be performed in light of highly variable context specific cut-offs, depending on different clinical scenarios and variable targets (i.e., predictors of weaning, extubation or noninvasive ventilation failure, muscle exhaustion, or titration of mechanical ventilation).

As future perspectives, new ultrasound applications have been proposed in the assessment of diaphragmatic activity, i.e., diaphragmatic tissue Doppler analysis, diaphragmatic shear wave elastography, and diaphragmatic speckle tracking analysis, evaluating diaphragmatic excursion kinetics, stiffness, and strain, respectively, during respiratory cycle [51]. The real impact of these tools needs to be addressed in large multicenter investigations.

### The abdomen

In case of unexplained shock associated to abdominal pain or not, bedside ultrasound assessment of the abdomen should be performed to diagnose possible underlying conditions. Furthermore, bedside sonographic reassessment of worsening patients suffered from blunt trauma should be executed to visualize previously undetected free fluids or to monitor the evolution of already known small amount of free fluid. Finally, abdominal ultrasound should be part of a systematic sonographic assessment of septic patients for searching the source of sepsis.

Despite significant evidence on the use of abdominal ultrasound in critical care [1], a structured formal training on how to perform abdominal ultrasound in the critically ill and how to certificate adequate competence has not yet been completely defined. Papers published in the last two decades from various scientific societies in different settings suggest that such an educational program should include formal didactic or web-based teaching of the basics of ultrasound, anatomy and pathology, and ultrasound-guided interventional procedures. The initial training should include laboratory training on healthy volunteers and simulators. Only after the trainee has met a minimum score of proficiency in the simulation phase a proctored clinical phase can be started, and then, provided there has been adequate progress along the learning curve, a personal learning phase with distant supervision may occur [66].

Recently, a consensus statement from the ESICM [1] included among the basic skills for intensivists the sonographic scanning from the epigastrium up to the mesogastrium at the level of umbilicus, both in a transverse and longitudinal plane, for ruling out or confirming a ruptured aortic abdominal aneurysm in case of unexplained shock, abdominal pain, pulsatile mass, or lower extremities emboli [67].

The minimal requirements for optimal imaging acquisition and interpretation are reported in Table 4.

**Table 4:** Abdominal ultrasound

Condition	Imaging acquisition	Anatomy, findings and measurements	Training and learning curve
Abdominal aortic aneurysm (AAA)	- Use a convex probe 3.5–5 MHz - Place the transducer perpendicular to the subcostal area below the xiphoid process with the notch toward the patient’s right and adjust depth to visualize the abdominal aorta (AA) - In this position, measure transverse diameters at the proximal, mid, and distal segments - Rotate transducer to the longitudinal plane with notch pointing toward the patient’s head - In this position, scan the AA in the longitudinal plane from origin (subcostal window) to bifurcation (1–2 cm below the umbilicus) by moving the probe caudally - Pay attention that the transducer is positioned parallel to the long axis of the AA - Measure maximal antero-posterior diameter of AA in proximal, mid (near renal arteries), and distal (above iliac bifurcation) segments - Adjust depth while carrying out the examination since AA becomes more superficial as it courses through the abdominal cavity If AA cannot be optimally visualized, try to apply a gentle pressure with transducer to displace bowel gas, or use lower transducer frequency	- In the transverse view both AA and inferior vena cava (IVC) can be recognized on either side of the spine; also, origins of renal arteries are visualized - AA is visualized in a cross section allowing measurement of the antero-posterior and side-to-side diameters - In the longitudinal view, AA is visualized in its long axis allowing measurement of the antero-posterior and cranial-to-caudal diameters - In this view also the two major proximal branches are visualized: celiac trunk and superior mesenteric artery Upper limit of normal antero-posterior diameter above 50 years - 26.6 mm in men and 23.6 mm in women for the proximal AA - 22.5 mm in men and 18.3 mm in women for the distal AA	- 15-day course - Formal or web-based teaching - Laboratory training on healthy volunteers and simulators - Minimum Passing Score - Learning Curve: 25 up to 50 examinations on average
Hydronephrosis	- Use a convex probe 3.5–5 MHz - The kidney should be scanned in both long and short axes - To obtain a long axis view of the right kidney, the transducer is placed along the right lower intercostal spaces on the mid-axillary line with the transducer directed posteriorly and the notch pointing towards the head of the patient Please note that, the transducer should be swept anteriorly to posteriorly and cephalad to caudad in order to image the entire kidney - To obtain a long axis view of the left kidney, the transducer is placed on the posterior axillary line along left lower intercostal spaces but more cephalad than the right kidney Please, note that to obtain a long axis view of the left kidney, a more posterior approach is required to avoid stomach or intestinal air - For the short axis view of both kidneys, the transducer is rotated 90° from the long axis with indicator pointing down - Thickness measurement should be made between the surface of the kidney and a point where the parenchyma reaches the renal sinus	- Hydronephrosis appears as anechoic area within the normally echogenic renal sinus. The degree of hydronephrosis is based on visual diagnosis and is graded as mild, moderate or severe - The longitudinal diameter of the kidney ranges from 9 to 12 cm - The normal thickness varies from 1.5 to 1.8 cm, with mean values greater in males	- 2-week course - 25 to 50 proctored examinations → 50 are needed to reach enough accuracy for grading the severity of hydronephrosis
Bladder evaluation	- Use a convex probe 3.5–5 MHz -The probe should be placed on midline in the suprapubic area with the indicator pointing toward patient’s head Then, the probe may necessitate to be angled slightly downward toward the pelvis until the bladder is visualized In the longitudinal scan antero-posterior and cranial-caudal diameters should be measured - The probe should be then rotated counterclockwise until the probe indicator is pointing to the patient’s right to obtain a transverse view and to measure the latero-lateral diameter	- US measurement of urine volume in the bladder allows to rule out bladder overdistension and to establish catheterization need - The three measured diameters should be used to calculate the bladder volume as follows: [Depth (mm) × length (mm) × width (mm)] × 0.7 = bladder volume (mL)	-5 to 10 proctored examinations
Acute renal failure (ARF)	-Color and Power Doppler allow to identify vessels at the level of the hilum and in the renal parenchyma - Intrarenal vessels are better assessed by transverse scans that allow a Doppler angle closer to 0° and, thus, a higher sensitivity in detecting slow flow in small vessels - Knobology: (a) assess pulse repetition frequency (PRF) 1.2–1.4 kHz, and (b) velocity of the waveform 25–50 cm/s	- The RI is given by the ratio between systolic peak and diastolic peak/ systolic peak, and the normal value is 0.58 ± 0.10 - Values > 0.70 are considered abnormal, although a major clinical significance is observed only for values > 0.80 which are correlated with a worse outcome	Not yet well defined for critical care setting
Gastric distension	Low-frequency (2–5 MHz) convex transducer Supine and right lateral decubitus position Semi-recumbent is an alternative if unable to turn lateral Transducer should be placed along the sagittal plane in the epigastrium, perpendicular to skin Landmarks to be observed: - Vertebral bodies - Long axis of abdominal aorta - Pancreas - Liver - Short axis of gastric antrum	Grade 0 - Empty antrum - “Bull’s-eye” appearance - Thick muscularis propriae layer - Low pulmonary aspiration risk Grade 1 - Fluid visible in right lateral decubitus only - Low pulmonary aspiration risk Grade 2 - Fluid visible in both supine and RLD - High pulmonary aspiration risk - Thick fluids - Distended antrum - Recently ingested with ‘frosted glass’ appearance - Later stages associated with hyperechoic, heterogeneous consistency - Highest pulmonary aspiration risk Calculation of antral cross‐sectional area (CSA) CSA (cm2) = antero-posterior diameter × craniocaudal diameter × *π*∕4 (1)	33 supervised examinations

Studies have reported that different physicians from various subspecialties and different levels of training can be able to identify the aorta using ultrasound with enough degree of accuracy. Hoffmann et al. have found that also inexperienced sonographers might achieve acceptable performance with an appropriate training requiring > 25 proctored examinations needed to ensure competency [68]. Nguyen et al. further confirmed that novices could be trained to sonographic diagnosis of abdominal aortic aneurysm after 15 days of training, consisting in both theoretical and practical components, the letter including a learning curve of 50 examinations on average [68]. Furthermore, training on technically difficult cases should be part of the credentialing process.

No significant data have been published on how many examinations are needed per year to maintain proficiency in the setting of critical care.

POCUS can be helpful in case of urosepsis, allowing to detect infected hydronephrosis (Table 4) [69].

When hydronephrosis is identified, both kidneys and the bladder should be evaluated [70]. In fact, bladder outlet obstruction usually presents with bilateral hydronephrosis, while unilateral hydronephrosis can be an early sign of disease. Ultrasound allows to estimate urine volume in the bladder, to rule out bladder overdistension and define the need of catheterization.

High value (> 0.77) of resistive index of the renal interlobar arteries assessed through pulse-wave color Doppler has been shown to predict adverse outcomes and renal failure progression in septic patients and after major or cardiac surgery [71].

Regarding sonographic diagnosis of hydronephrosis, Sibley et al. have found that a learning curve consisting in 25 examinations at least may be enough to achieve proficiency [72]. On the other hand, Herbst and coll. have proved that a good accuracy in sonographic diagnosis of hydronephrosis can be achieved after a 2-week course including no less than 50 examinations as the main learning curve [73]

Training for evaluation of Doppler-derived renal resistivity index (RI) is not defined yet and requires further evidence.

In patients with acute abdominal pain, unexplained shock or septic shock, abdominal ultrasound is useful to assess differential diagnosis and to define the need of further radiological exams to identify possible underlying conditions. However, in this context, abdominal ultrasound performed bedside by intensivists reaches its maximum effectiveness only if clinically driven.

The presence of previously undetected abdominal free fluids in a symptomatic patient suggests acute abdomen needing further diagnostic exams and eventually surgery consultation [74].

Increased echogenicity of the peritoneal stripe with multiple reverberation artifacts may be a sign of free intraperitoneal air due to perforation [74].

A dilated small bowel loop with reduced or absent peristalsis, characterized by the “to-and-fro” movement of the intestinal content, also associated to gastric distension suggests bowel occlusion [74].

In a context of sepsis or septic shock and jaundice associated to elevated liver function tests, sonographic appearance of the gallbladder characterized by thickened walls (> 3 mm) associated or not to fluid peripheral collections, gallbladder dilation (length > 4 cm), hydrops in conjunction with large gallbladder stones, and presence of biliary sludge or pus are signs of acute cholecystitis [74].

No robust evidence is still available regarding training and learning curve for abdominal ultrasound performed in a critical care context by intensivists. Further studies are needed.

Ultrasound for detection of free fluids in the acute traumatic abdomen is well known as clinical practice since part of focused assessment with sonography for trauma (FAST) examination recommended by the American College of Surgeons in the Advanced Trauma Life Support Course.

After the first assessment in the Emergency Room, this kind of examination should be repeated also during ICU stay as a monitoring tool or in case of unexplained hemodynamic instability allowing to detect eventually previously undetected free fluids collection or an increase in already known small amount of free fluid.

With the aim of establishing the competency in detecting intraperitoneal free fluids at FAST examination, 10 examinations might not be sufficient [75]. The learning curve starts to flatten at 30 examinations, while after 50 examinations at least high accuracy is reached [76]. These differences among studies are related to the fact that all these studies have attempted to define competency by different methods of analysis.

Bowel dysfunction, associated to vomiting and abdominal distension may be common in the critically ill, especially in patients who underwent major abdominal surgery. Gastric ultrasound allows both a qualitative and quantitative assessment of the stomach allowing to rule out postoperative ileus and to define risk of aspiration [77, 78].

When properly clinically driven, gastric ultrasound reaches high sensitivity (1.0), specificity (0.975), positive predictive value (0.976), and negative predictive value (1.0) [79].

Arzola and coworkers have found that 33 supervised gastric scans are required to achieve a 95% accuracy in qualitative assessment of gastric content [80]

Finally, in septic shock, ultrasound allows to detect abscesses, necrotizing fasciitis, cellulitis, or thrombophlebitis as potential underlying source of sepsis with good accuracy [81, 82]. Berger et al. have found that only a minimal training consisting in a 2-day course is required to reach enough accuracy for soft tissue infections diagnosis [83].

### The vessels

The global use of ultrasound is recommended to assist all steps of ultrasound vascular access device placement: (a) rational choice of appropriate vein and proper approach; (b) prevention of primary malposition; (c) ruling out of respiratory complications; (d) ruling out of late complications (i.e., catheter-related thrombosis, fibrin sleeve) [66] (Table 5).

**Table 5:** Vascular ultrasound: expected core competencies and learning goals

Procedure/condition	Imaging acquisition	Main findings/procedures	Training and learning curve
Ultrasound-guided vascular access device placement	1. Preliminary assessment	- Sonographic assessment of all possible venous options to choose the easiest and the safest approach	- Formal didactic or web-based teaching - Simulation lab: (a) healthy volunteers; (b) simulators (biological or computer-based) - Minimum passing score - 5 observed procedures of each kind - 5 proctored procedures of each approach - 30 ultrasound-guided vascula access placement of any kind within 1 year
	2. Ultrasound-guided puncture	- Suggested protocols for assessment: RaCeVA for cervical and thoracic vessels [84]; (b) RaFeVA for lower limbs and groin vessels [85]; RaPeVA for deep peripheral vessels [86]	
	3. Ultrasound-based tip navigation		
	4. Ultrasound-based tip location	- Follow the needle tip visualizing it into the vessel’s lumen	
	5. Lung ultrasound to rule out possible respiratory mechanical complications	- Follow wires, dilator or microintroducer, and catheters while advancing through vessels tributary of the superior or inferior vena cava [87]	
	6. Compressive ultrasound to rule out catheter-related thrombosis in case of signs and symptoms eventually associated to catheter’s malfunctioning	- Subcostal 4-chamber, subcostal bicaval or apical 4-chamber views to identify catheter’s tip at the junction between right atrium and superior or inferior vena cava [87, 88] - Perform lung ultrasound to rule out pneomothorax or hemothorax after a difficuly venipuncture or in case of sudden and otherwise unexplained worsening of respiratory or hemodynamic conditions [66] - Perform compressive ultrasound to exclude a clinically suspected catheter-related thrombosis (CRT) exploring all vessels starting from catheter’s entry point [66] - Ultrasound allows to distinguish between a true CRT and the fibrin sleeve	
Deep vein thrombosis (DVT)	Compressive ultrasound (CUS) in case of suspected deep vein thrombosis or pulmonary embolism	- 7.5–15 MHz linear probe - CUS of common femoral vein at the groin and its conluence with great saphenous vein - CUS of superficial femoral vein at the mid-tight - CUS of popliteal vein in the popliteal fossa	2 to 10-h course 25 to 50 proctored examinations

General curriculum for ultrasound-guided vascular access procedures should consist of didactic lectures or web-based teaching; laboratory training including simulation training; a clinical phase that includes both closely supervised and distant supervised learning. Following ESAIC guidelines, each trainee should observe and then perform, under supervision, 5 procedures of each kind of approach. Finally, perioperative use of ultrasound-guided for vascular access guidelines from European Society of Anesthesiology and Intensive care (ESAIC) suggests at least 30 US-guided procedures of any type in a 12-month period for completion of competency-based training, as personal learning curve [66]

In addition, compressive ultrasonography of common femoral vein at the groin, superficial femoral vein at the mid-thigh, and popliteal vein in the popliteal fossa allows to rule out deep vein thrombosis with good accuracy.

## Conclusions

**Fig. 5:**
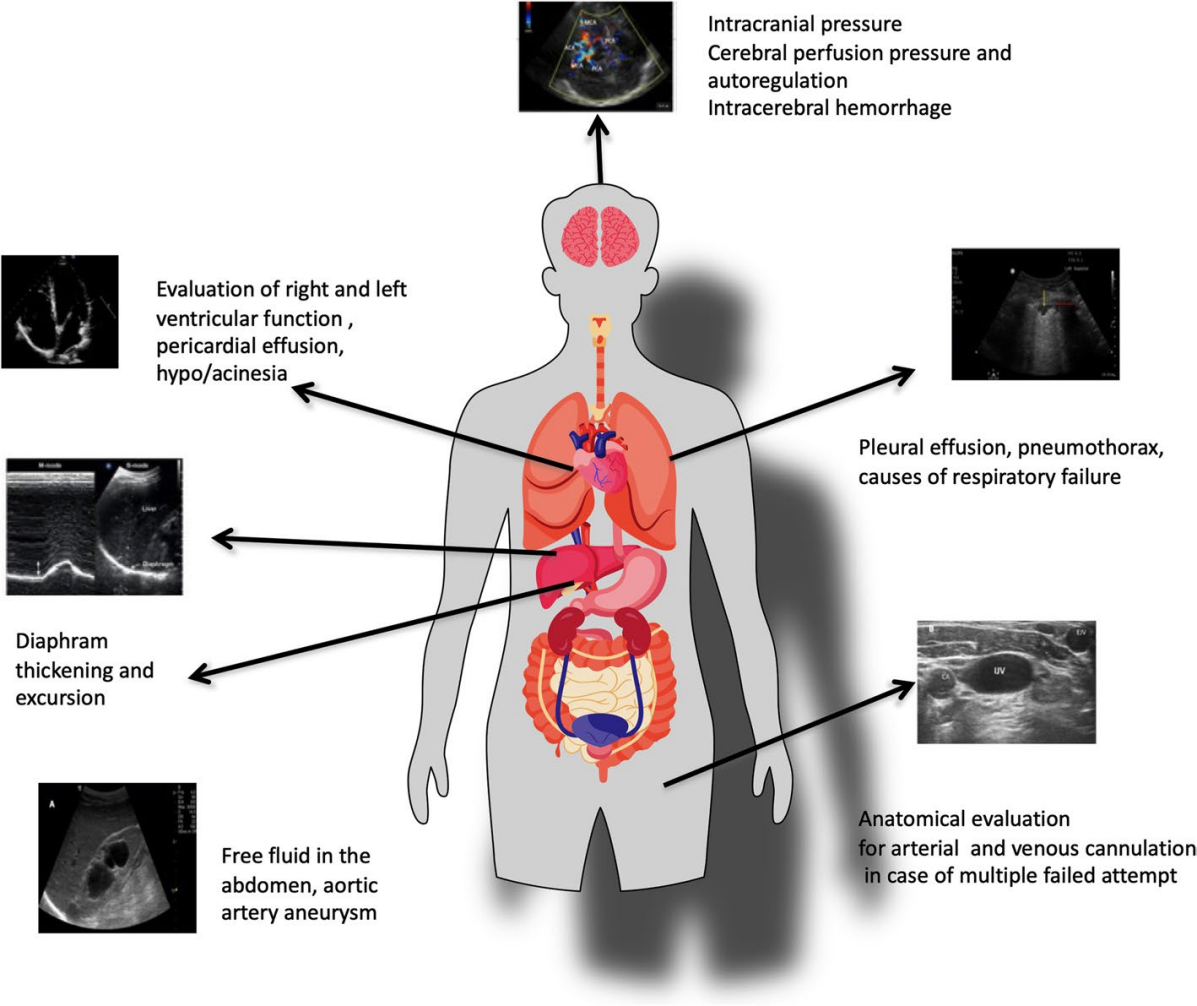
Head to toe ultrasound assessment in critically ill patients. Head to toe ultrasound assessment in critically ill patients for brain, lung, heart, diaphragm, abdomen, and vessels with the main clinical applications is represented

Head to toe ultrasonography is becoming a fundamental skill for the bedside assessment of patients in the ICU (Fig. 5).

A standardized training program and certification is required to improve physician’s knowledge and patients care in these settings.

## Data Availability

Not applicable.

## Abbreviations

aSAH: Aneurysmal subarachnoid hemorrhage
BUS: Brain ultrasound
CA: Cerebral autoregulation
CBF: Cerebral blood flow
CT: Computed tomography
COVID-19: Coronavirus 2019 disease
CCE: Critical care echocardiography
US: Critical care ultrasonography
DSA: Digital subtraction angiography
ESAIC: European Society of Anesthesiology and Intensive care
ESICM: European Society of Intensive Care
FAST: Focused assessment with sonography for trauma
ICU: Intensive care unit
ICP: Intracranial pressure
POCUS: Point-of-care ultrasound
IMV: Invasive mechanical ventilation
RI: Resistivity index
TCCD: Transcranial color-coded duplex ultrasonography
TEE: Transesophageal echocardiography
TTE: Transthoracic echocardiography
